# Expectant management for umbilical artery thrombosis in monochorionic diamniotic twin pregnancies: a case report

**DOI:** 10.1186/s12884-023-05834-9

**Published:** 2023-07-14

**Authors:** Qilin Wang, Yanping Zhang, Rong Zhou

**Affiliations:** 1 Department of Obstetrics and Gynecology, West China Second University Hospital, Sichuan University, Chengdu, 610041 Sichuan province China; 2 Key Laboratory of Birth Defects and Related Diseases of Women and Children, Sichuan University of Ministry of Education, Chengdu, 610041 Sichuan P.R. China

**Keywords:** Umbilical artery thrombosis, Twin pregnancy, Monochorionic, Perinatal outcome, Fetal therapy

## Abstract

**Background:**

Thrombosis of one of the umbilical arteries is a rare complication of pregnancy and is associated with adverse pregnancy outcomes, including stillbirth and intrauterine growth restriction. Although extremely rare, umbilical artery thrombosis (UAT) in monochorionic diamniotic twins is difficult to diagnose prenatally and manage. UAT has a poor prognosis and is associated with an increased perinatal mortality rate. In most previous cases, emergency cesarean section was performed or intrauterine fetal death occurred at the time of UAT diagnosis. **Case presentation**: Herein, we report an extremely rare case of sequential UAT in monochorionic diamniotic twins diagnosed via ultrasound at 29^+ 5^ weeks of gestation in a 34-year-old woman. Following expectant management with intensive monitoring for 16 days, two healthy infants were delivered through an emergency cesarean section. UAT in both fetuses was confirmed by pathological examination. The mother and twins described in this case underwent long-term follow-up and are currently in good health without any complications.

**Conclusions:**

Based on our experience, we suggest that expectant management should be undertaken as long as the mother and infants are stable on ultrasonographic scans and are closely monitored. When UAT is suspected, we believe that the best delivery time should be determined by considering complaints of unusual fetal movements, non-stress test evidence, gestational age, amniotic fluid volume, and blood flow in the umbilical artery, middle cerebral artery, and ductus venosus. Obstetricians should ensure that the patients and their families are clearly informed about all potential risks of expectant management for UAT.

## Background

Umbilical artery thrombosis (UAT) is a rare but serious complication of pregnancy that is associated with increased perinatal morbidity and mortality rates, as well as adverse pregnancy outcomes, such as intrauterine growth restriction, intrauterine death, stillbirth, and neonatal death. The reported incidence of UAT ranges from 0.0025 to 0.045% of gestations, and it has a poor prognosis [1]. Nonetheless, precise prenatal diagnosis and clear management of UAT are not yet fully understood. UAT in monochorionic diamniotic twins is extremely rare, and no case of prenatal diagnosis has been reported. Herein, we report a case of UAT in monochorionic diamniotic twins prenatally diagnosed via ultrasonography.

## Case Presentation

A 34-year-old primipara (gestation, 3; parity, 0; abortion, 2) who had spontaneously conceived presented to a local hospital for routine prenatal transvaginal ultrasonography. The patient had undergone induced abortions twice at 7 + weeks of gestation 2 and 7 years previously, and had no history of recurrent miscarriage or antiphospholipid syndrome. Her medical, surgical, and family histories were unremarkable. At 7 + 2 weeks of gestation, a monochorionic twin pregnancy was diagnosed. At 12 + 2 weeks, ultrasonography showed a monochorionic diamniotic twin pregnancy with nuchal translucency thicknesses of 1.7 and 2.0 mm. At 22 weeks, two umbilical arteries and one umbilical vein were detected by conventional color Doppler ultrasound in both twins, with umbilical cords marginally inserted into the placenta. No structural anomalies were observed in either twin. The results of oral glucose tolerance testing were negative. No abnormalities were identified until 28 weeks’ gestation.

During the third trimester, a routine ultrasound of fetus A at the local hospital showed a single right umbilical artery, while the other umbilical cord carried a hyperechoic segment. This finding raised suspicion of spontaneous intrauterine UAT in fetus A. At 29 + 5 weeks of gestation, the patient was referred to our hospital, which is an academic tertiary referral care center. There were no obvious abnormalities with respect to maternal blood test results (including coagulation function and D-dimer assay), maternal blood pressure, amniotic fluid volume, and fetal growth. Color Doppler examination indicated normal blood flow in the umbilical artery, middle cerebral artery, and ductus venosus in the twins. The non-stress test for fetal heart rate monitoring showed responsive results.

The reason for the patient’s first admission at 29 + 5 weeks of gestation was expectant management under strict fetal observation to prolong the gestational age as much as possible, improve the adverse pregnancy outcomes, and avoid extremely preterm infant birth. Our expectant management included three components, as follows: (1) daily intermittent oxygen inhalation (twice daily for 30 min) and intensive monitoring of the twins, including fetal heart rate auscultation every 4 h, electronic fetal heart rate monitoring twice daily, and daily assessment of fetal movements; (2) ultrasound screening of the amniotic fluid volume and blood flow in the umbilical artery, middle cerebral artery, and ductus venosus of the twins every 3 days to ensure the twins’ well-being, with fetal growth biometry conducted every week until delivery; and (3) assessment of the maternal risk of thrombosis. The maternal risk factors for thrombosis were assessed repeatedly at admission to the hospital and after delivery. This patient had only one risk factor: multiple gestation before delivery. Before admission, the patient had a clerical job and walked approximately 7,000 to 8,000 steps to work each day. At 31 + 2 weeks of gestation, the patient experienced sudden a gush of fluid and irregular uterine contractions. Doppler ultrasound showed that the amniotic fluid depth of fetus A was reduced from 7.5 to 3.8 cm, while that of fetus B was maintained at approximately 6.7 cm. Preterm premature rupture of membranes of fetus A was diagnosed. Despite negative results on detailed examinations, including cervical secretion culture and group B streptococcus culture, and exclusion of chorioamnionitis, the patient was at high risk for preterm labor. To manage the situation, bed rest was implemented, and the patient was administered a range of tocolytic therapies, including ritodrine to suppress uterine contractions, cefoxitin to prevent maternal and neonatal infections, and corticosteroids to accelerate fetal lung maturity.

**Fig. 1:**
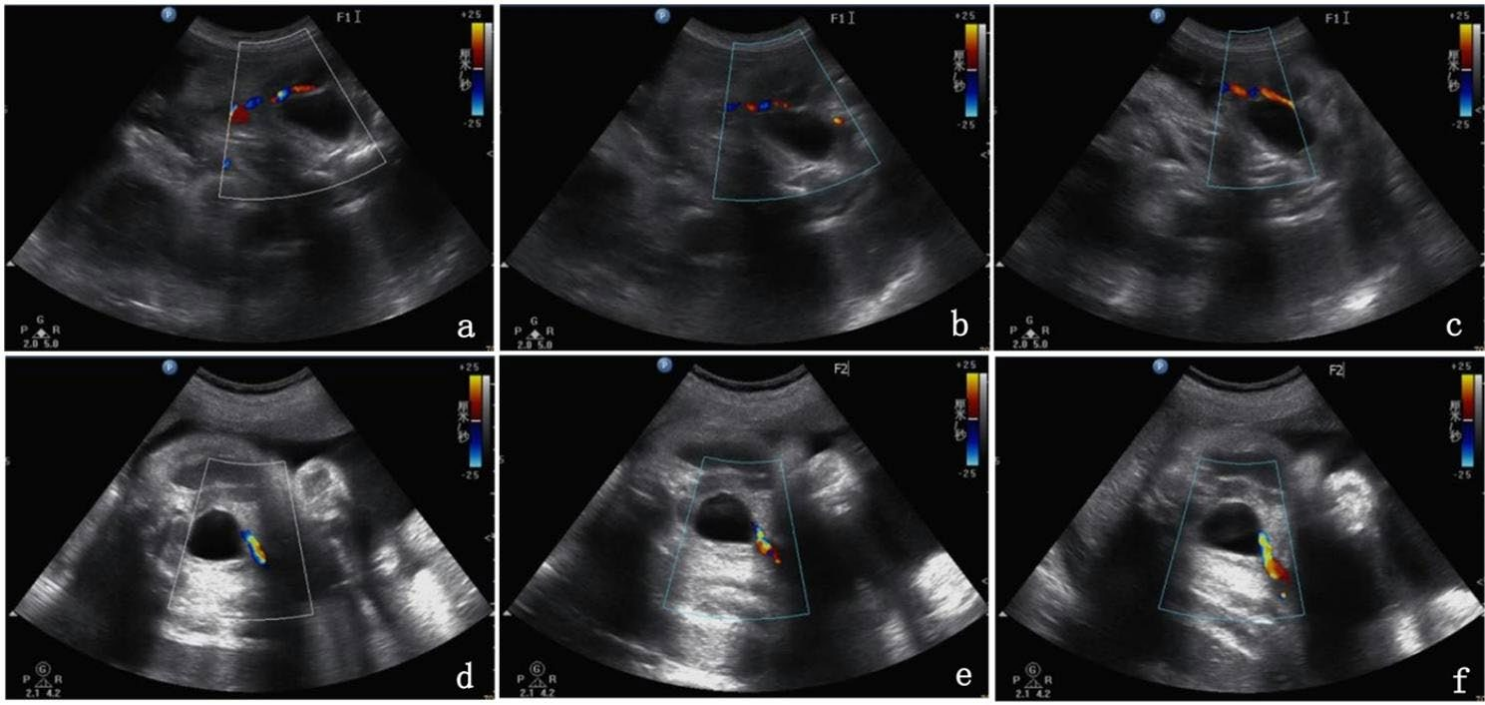
Cross-sectional views of ultrasound images at the level of the twins’ bladder at 31 + 6 weeks. The SUA blood flow images was shown on both sides of bladder of fetus A **(a)** and fetus B**(d)**. Amidst continuous and dynamic umbilical artery blood flow observation and changing fetal position, there was still only one umbilical artery is visualized at the level of the bladder of fetus A **(b,c)** and fetus B **(e,f)**. UAT should be seriously considered when only one umbilical artery is found for the first time in the third trimester

**Fig. 2:**
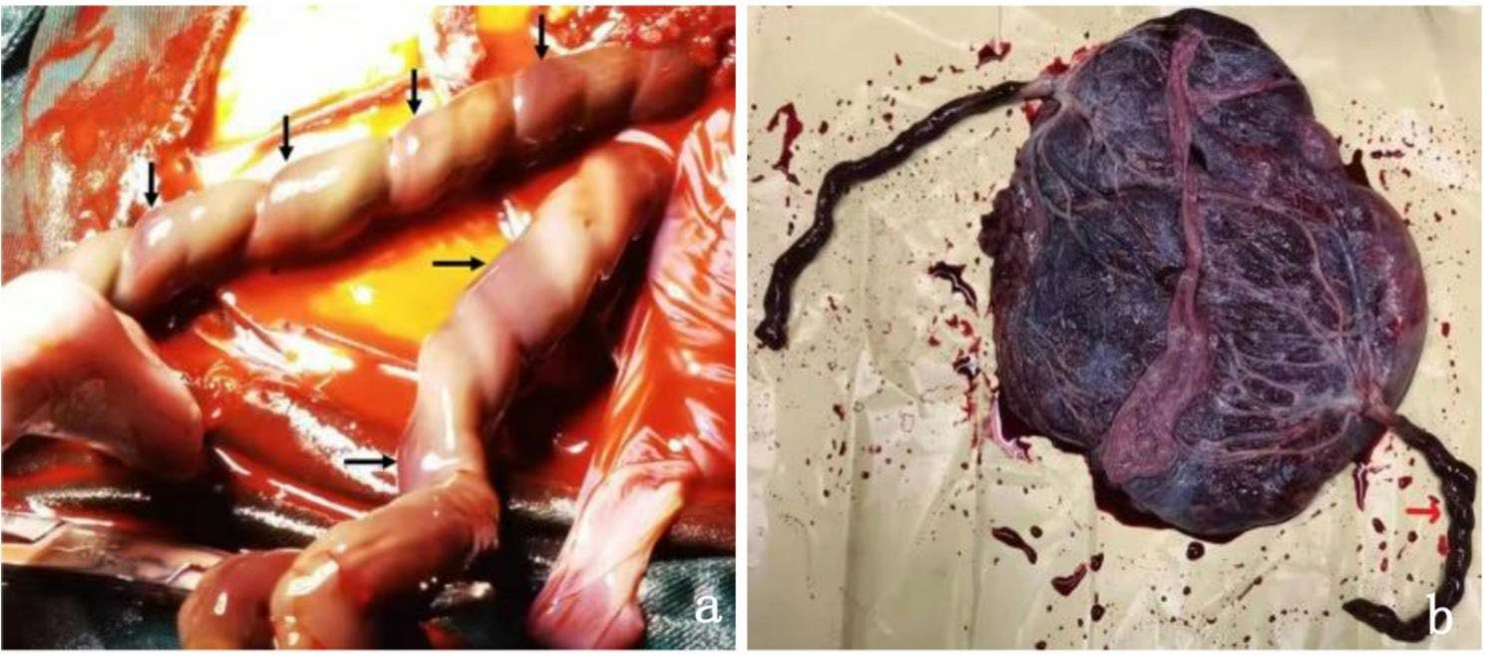
Clinical features of the patient. During the surgery, UAT (black arrow) was observed in both fetuses **(a)**. Both umbilical cords were edematous, rigid, and marginally inserted into the placenta **(b)**. The cord was extremely twisted 20 times in fetus B (red arrow)

**Fig. 3:**
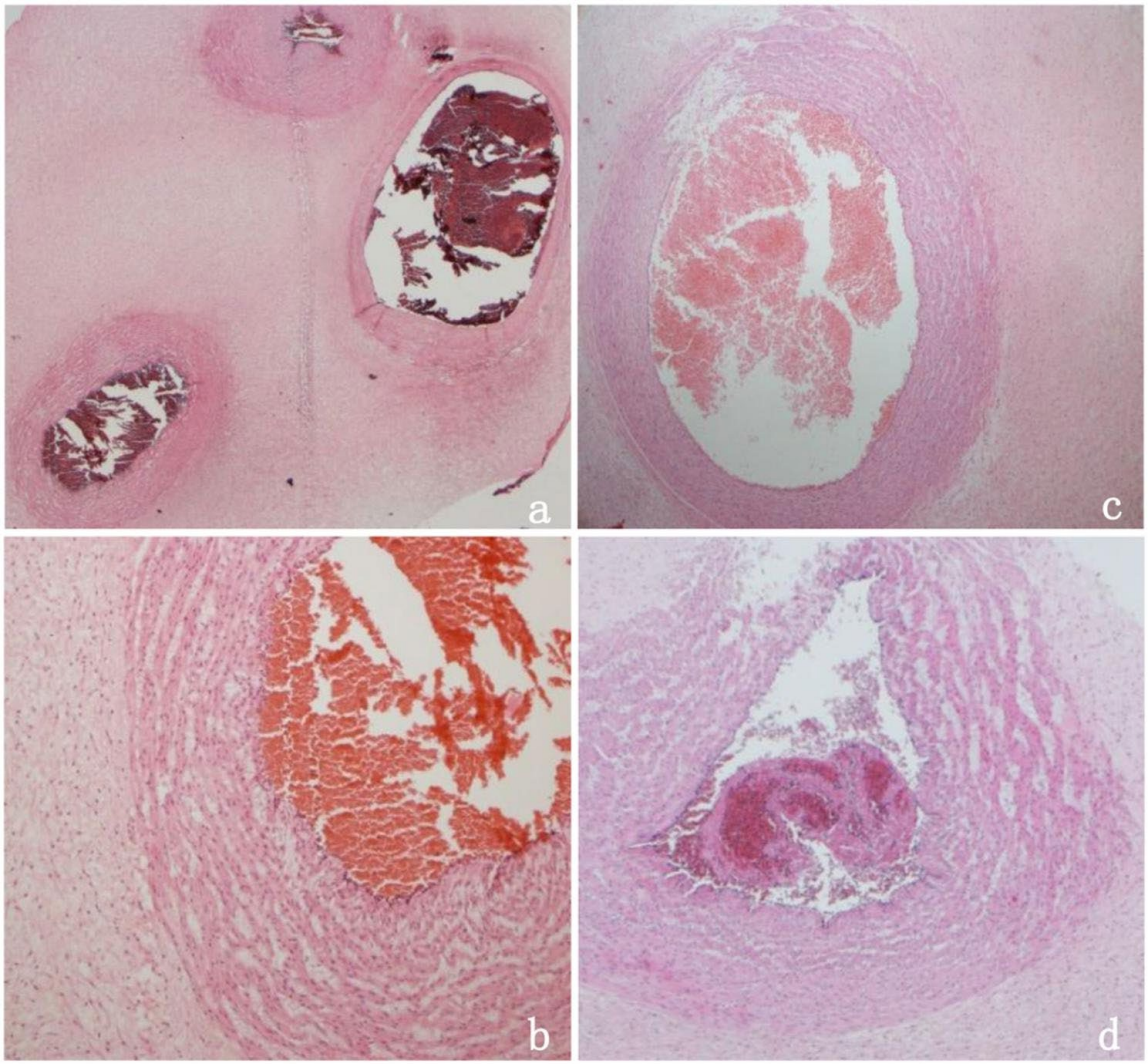
Histology of the twin’s umbilical cord. Two umbilical arteries and one umbilical vein were detected of fetus A. Thrombosis of umbilical artery of fetus A, hematoxylin and eosin×25 magnification **(a)**; ×100 magnification **(b)**. Thrombosis of umbilical artery of fetus B, hematoxylin and eosin×50 magnification **(c)**. A white thrombus was found in the umbilical artery of fetus B, hematoxylin and eosin×50 magnification **(d)**

Four days later, at 31 + 6 weeks, while following the UAT in fetus A, an ultrasound examination of fetus B revealed a single umbilical artery (SUA) at the level of the bladder **(**Fig. 1a, d**)**. As the SUA could be seen during continuous dynamic observation and changing fetal position, the possibility of UAT was considered **(**Fig. 1b, c, e, f**)**. However, Doppler flow in both twins did not show any abnormalities. At 32 weeks, the patient reported an increase in fetal movements. Electronic fetal heart rate monitoring showed prolonged spontaneous decelerations, in which the heart rate of fetus A remained between 70 and 90 beats per minute for > 2 min. UAT was highly suspected to be the cause of prolonged spontaneous decelerations in fetus A. After obtaining informed consent from the patient and her family, an emergency cesarean section was performed, and two healthy infants were delivered after 16 days of expectant management. During the surgery, UAT was observed in one umbilical artery in both fetuses **(**Fig. 2a**)**. In addition to the umbilical cords, the placenta was sent for pathological examination. Gross examination revealed two edematous umbilical cords that were dark red in color, appeared rigid, and were marginally inserted into the placenta; the lengths of the umbilical cords were approximately 52 and 45 cm, respectively **(**Fig. 2b**)**. The umbilical cord of fetus B was extremely torsional, with 20 laps. Histopathological examination did not demonstrate any thrombi or infarction of the fetal or maternal surfaces of the placenta. The cut surface showed one vein and two arteries in each umbilical cord, with one of the arteries displaying blood flow blockage. Pathological examination confirmed UAT in one of the arteries in both fetuses **(**Fig. 3a, b, c, d**)**.

Female infant A was born with a birth weight of 1960 g, with 1-, 5-, and 10-min Apgar scores of 8, 9, and 9, respectively, and umbilical arterial pH of 7.413. In contrast, female infant B was born with a birth weight of 1640 g, with 1-, 5-, and 10-min Apgar scores of 8, 9, and 9, respectively, and umbilical arterial pH of 7.385. Due to their prematurity, both twins were transferred to the neonatal intensive care unit and required respiratory support. Fortunately, the twins experienced good postnatal development without any serious complications and were discharged from the hospital on the 24th day of life, weighing 2080 and 2360 g, respectively. The cesarean section was an additional risk factor for venous thromboembolism after delivery in this patient. Low-molecular-weight heparin (4,100 IU every 24 h) was injected subcutaneously 24 h after the cesarean section until discharge. During close follow-up of the patient and infants for up to 18 months, no thrombotic events were observed in the mother, and there were no abnormalities in the physical, mental, and intellectual development of the twins. To date, they are alive and thriving.

## Discussion and conclusions

Umbilical vessel thrombosis is a rare condition that is insidiously associated with serious complications of pregnancy, such as fetal distress, stillbirth, and hypoxic-ischemic encephalopathy. Umbilical cord thrombosis has been reported to occur in approximately 1 out of 1300 deliveries, 1 out of 1000 postnatal autopsies, and 1 out of 250 high-risk pregnancies [2]. Although UAT rarely occurs, it has a worse prognosis. Furthermore, previous studies have reported that this disease mainly occurs in singleton pregnancies. UAT in monochorionic diamniotic twins is rare, with no reports of prenatal diagnosis, although good outcomes have been reported.

Previous studies have suggested that congenital umbilical cord dysplasia, infections, increased maternal blood glucose levels, and maternal coagulation status are related to the occurrence and development of thromboembolism [3]. The etiology and specific mechanism of fetal UAT remain unknown due to the absence of reliable research data; nonetheless, this may be explained by Virchow’s hypothesis for thrombosis. Three key factors are responsible for thrombosis: hypercoagulability, blood flow stasis, and endothelial injury [4]. Using logistic regression, Wu et al. recently revealed that maternal gestational diabetes mellitus and fetal umbilical cord abnormalities were also independent risk factors for UAT in their retrospective cohort study [5]. However, the study had major limitations: first, the data collected were based on retrospective studies and registry data, and second, the generalizability to a broader population with UAT remains unknown. Most reported cases of UAT were associated with umbilical cord abnormalities, which can easily cause umbilical cord compression, leading to a reduction in blood flow and consequent thrombosis [6–8]. In this case, prolonged bedrest due to preterm premature rupture of membranes at 31 + 2 weeks and multiple pregnancies aggravated the hypercoagulable state. The twins’ umbilical cords were marginally inserted into the placenta; fetus B had a cervical umbilical cord loop, and the umbilical cord of fetus B was extremely torsional in 20 laps. Premature rupture of membranes long before term can be infectious. Although there was no positive culture result in this case, vascular endothelial injury might have been present, leading to the subsequent development of UAT in fetus B.

The incidence of SUA varies from 0.2 to 0.87%, and it is more common in women with multiple pregnancies [9]. The most widely accepted underlying explanations for an SUA include primary agenesis of one umbilical artery, later thrombotic atrophy of one umbilical artery, or persistence of the original single allantoic artery in the body stalk [10]. Neonates with SUAs are at a high risk for congenital anomalies and chromosomal abnormalities. The most common congenital anomalies associated with SUAs are renal anomalies, followed by cardiovascular and musculoskeletal anomalies [11]. In our case, non-invasive prenatal testing of the patient indicated low risk. At 22 weeks, no structural anomalies were observed on conventional color Doppler ultrasound and fetal echocardiography in either twin. Based on the ultrasound results of this patient, UAT should be considered when color Doppler ultrasound during the third trimester indicates an SUA.

The characteristic ultrasound finding for UAT is an appearance similar to that of an orange grabbed by one hand, owing to an occluded artery that is parallel to the remaining artery and surrounded by the umbilical vein [7]. However, in our case, this sign was not observed. Instead, the ultrasound of fetus A showed a single right umbilical artery, whereas the other umbilical cord displayed a hyperechoic segment. At 31 + 6 weeks, ultrasound examination of fetus B revealed an SUA with a cervical umbilical cord loop. This patient was referred to our hospital at 29 + 5 weeks of gestation, which caused difficulties in diagnosing UAT. Ultrasonography is an essential and useful tool for prenatal diagnoses. UAT should be suspected when two umbilical arteries become one or a single artery is observed on ultrasonography. The number of umbilical arteries can be confirmed by evaluating blood flow at the level of the fetal bladder, and current and previous ultrasound images of umbilical cord vessels should be compared. Highly experienced obstetricians and sonographers are required to make an accurate prenatal UAT diagnosis. Ultimately, the diagnosis of UAT is confirmed by pathological examination.

Previous studies have reported that emergency cesarean section is commonly performed upon confirmation of UAT diagnosis during the third trimester [3]. When UAT is found at or near term, emergency cesarean section with monitoring of abnormal fetal heart rate is preferred. However, when UAT accompanied by fetal growth restriction is detected in the second trimester, pregnancy management becomes challenging [12]. Obstetricians may hesitate to intervene when managing fetuses with UAT and often rely on their own experience and the fetal status to determine treatment options [13].

In the study by Wu et al., it was found that the expectant management group did not have worse fetal outcomes compared to the urgent treatment group when current screening and therapeutic strategies were implemented [5]. Therefore, fetal protection should be considered for fetuses with very low gestational age, whereas urgent delivery is preferred for fetuses with higher gestational age to avert unnecessary fetal loss. For patients with UAT and good maternal and fetal status, particularly the preterm group with an urgent need to extend the pregnancy, expectant management with ultrasound screening and close fetal monitoring may be an alternative to emergent delivery [14], as seen in our case.

Owing to the rarity of fetal UAT and the limited research data, there is currently no consensus on the treatment strategy for fetal UAT. Herein, we share our experience with UAT in a monochorionic diamniotic twin pregnancy. Expectant management with intensive monitoring successfully rescued both twin fetuses with UAT. The patient underwent emergency cesarean section at 32 weeks of gestation after 16 days of expectant management with intensive monitoring due to acute fetal distress indicated by the non-stress test. Because of dynamic monitoring and timely intervention, our case had a good prognosis without complications. At long-term follow-up, the twin sisters exhibited no neurological or cognitive impairments and are in good health. Based on this case, expectant management requires close observation of clinical symptoms (e.g., fetal heart rate, fetal movements) and ultrasound indices, such as fetal growth, amniotic fluid volume, and blood flow of the umbilical artery, middle cerebral artery, and ductus venosus, in addition to electronic fetal heart rate monitoring. Patients should be hospitalized for expectant treatment. When we encounter presentations of fetal intrauterine distress, an emergency cesarean section can be performed immediately, thus avoiding adverse perinatal outcomes such as stillbirth.

To our knowledge, expectant management may not necessarily be acceptable to patients with UAT, and sudden fetal death can still occur without foretelling signals. Obstetricians should ensure that the patients and their families are clearly informed about all potential risks of expectant management of UAT, and written informed consent should be acquired.

## Data Availability

All data generated or analysed during this study are included in this published article. The datasets used and/or analyzed during this study are available from the corresponding author on request.

## Abbreviations

UAT: Umbilical artery thrombosis
SUA: Single umbilical artery

## References

[1] Kitano T, Ohgitani A, Takagi K, et al. A case of severe neonatal asphyxia due to umbilical artery thrombosis. J Obstet Gynaecol. 2018 Nov;38(8):1164–5. 10.1080/01443615.2017.1404012 29514532

[2] Heifetz SA (1988). Thrombosis of the umbilical cord: analysis of 52 cases and literature review. Pediatr Pathol.

[3] Zhu Y, Beejadhursing R, Liu Y. 10 cases of umbilical cord thrombosis in the third trimester. Arch Gynecol Obstet. 2021 Jul;304(1):59–64. 10.1007/s00404-020-05910-x 33389094

[4] Malone PC, Agutter PS. The aetiology of deep venous thrombosis. QJM. 2006 Sep;99(9):581–93. 10.1093/qjmed/hcl070 16905749

[5] Wu X, Wei C, Chen R et al. Fetal umbilical artery thrombosis: prenatal diagnosis, treatment and follow-up. Orphanet J Rare Dis 2022 Nov 12;17(1):414. 10.1186/s13023-022-02563-8 PMC9652808 36371215

[6] Oliveira GH, Dias Cde M, Vaz-Oliani DC, et al. Intrauterine thrombosis of umbilical artery - case report. Sao Paulo Med J. 2016 Jul-Aug;134(4):355–8. 10.1590/1516-3180.2016.00081203 PMC10876343 27276083

[7] Tanaka K, Tanigaki S, Matsushima M (2014). Prenatal diagnosis of umbilical artery thrombosis. Fetal Diagn Ther.

[8] Klaritsch P, Haeusler M, Karpf E et al. Spontaneous intrauterine umbilical artery thrombosis leading to severe fetal growth restriction. Placenta 2008 Apr;29(4):374–7. 10.1016/j.placenta.2008.01.004 18289672

[9] Vasanthalakshmi GN, Pushpalatha T, Mehta P (2012). Single umbilical artery and pregnancy outcomes: cause for concern. J S Asian Fed Obstet Gynaecol.

[10] Gornall AS, Kurinczuk JJ, Konje JC. Antenatal detection of a single umbilical artery: does it matter? Prenat Diagn. 2003 Feb;23(2):117–23. 10.1002/pd.540 12575017

[11] Murphy-Kaulbeck L, Dodds L, Joseph KS, et al. Single umbilical artery risk factors and pregnancy outcomes. Obstet Gynecol. 2010 Oct;116(4):843–50. 10.1097/AOG.0b013e3181f0bc08 20859147

[12] Wei J, Li Q, Zhai H. Umbilical artery thrombosis diagnosed at different gestational ages and fetal outcomes: a case series. BMC Pregnancy Childbirth 2021 Nov 22;21(1):788. 10.1186/s12884-021-04264-9 PMC8607721 34809600

[13] Li H, Qufeng W, Wei W, et al. Umbilical artery thrombosis: two case reports. Med (Baltim). 2019 Nov;98(48):e18170. 10.1097/MD.0000000000018170 PMC6890325 31770267

[14] Han C, Dong K, Jia Z, et al. Expectant management for umbilical artery thrombosis: a report of two cases and literature review. J Matern Fetal Neonatal Med. 2022 Dec;35(25):9296–8. 10.1080/14767058.2022.2029398 35086411

